# Proteasome Activation as a Therapeutic Strategy to Rescue Cognitive Decline in Aging and Alzheimer’s Disease

**DOI:** 10.1002/alz70861_108715

**Published:** 2025-12-23

**Authors:** Andrew Pickering, Maria Gaczynska, Pawel Osmulski, Karl Rodriguez

**Affiliations:** ^1^ University of Texas Health Houston, Houston, TX USA; ^2^ UT Health Science Center San Antonio, San Antonio, TX USA; ^3^ The University of Texas Health Science Center at San Antonio, San Antonio, TX USA

## Abstract

**Background:**

Declines in proteasome activity are a shared feature of aging and Alzheimer’s Disease (AD), particularly in the hippocampus. These impairments disrupt key neuronal functions including synaptic plasticity and memory formation. β‐amyloid (Aβ) oligomers have been shown to inhibit proteasome activity and destabilize its structure. Despite its central role in maintaining neuronal proteostasis, the proteasome remains an underexplored target for therapeutic intervention in AD and age‐associated cognitive decline.

**Method:**

We evaluated the therapeutic potential of proteasome activation in multiple models. A transgenic mouse model with neuron‐specific overexpression of the proteasome subunit PSMB5 (NSE‐PSMB5) was used to assess cognitive resilience during aging and in AD (hAPP‐J20) models. We also tested two proteasome‐activating peptidomimetics, TAT1‐DEN and TAT1‐8,9TOD, for their capacity to enhance proteasome function in vitro and in vivo. Behavioral outcomes were assessed using Morris water maze, Y‐maze, and object recognition assays in aged and AD‐model mice. Parallel studies were conducted in Drosophila and cell culture models expressing Aβ.

**Results:**

Aging and Aβ pathology caused significant reductions in both 20S and 26S proteasome activity and disrupted their structural integrity. PSMB5 overexpression preserved proteasome activity and mitigated age‐ and Aβ‐associated deficits in spatial memory and learning. Acute treatment with TAT1‐DEN restored hippocampal proteasome function and significantly improved cognitive performance in aged and AD‐model mice. In Drosophila, proteasome activation delayed mortality and reversed memory impairments. Mechanistic studies showed that Aβ oligomers impaired 20S gate conformation and promoted 26S disassembly—effects reversed by proteasome agonists. Proteasome activation also enhanced degradation of APP and reduced Aβ levels.

**Conclusion:**

Our findings demonstrate that proteasome dysfunction is a key contributor to cognitive decline in aging and AD. Both genetic and pharmacological augmentation of proteasome activity restore proteasome function and reverse cognitive impairments. These results support the proteasome as a viable therapeutic target for combating age‐ and AD‐related neurodegeneration.

